# Incidence of carditis and predictors of pacemaker implantation in patients hospitalized with Lyme disease

**DOI:** 10.1371/journal.pone.0259123

**Published:** 2021-11-03

**Authors:** Uwajachukwumma A. Uzomah, Guy Rozen, Seyed Mohammadreza Hosseini, Ayman Shaqdan, Pablo A. Ledesma, Xuejing Yu, Pegah Khaloo, Jennifer Galvin, Leon M. Ptaszek, Jeremy N. Ruskin

**Affiliations:** Cardiac Arrhythmia Service, MGH Heart Center, Massachusetts General Hospital, Boston, MA, United States of America; Fondazione IRCCS Policlinico San Matteo, ITALY

## Abstract

**Background:**

Lyme carditis, defined as direct infection of cardiac tissue by Borrelia bacteria, affects up to 10% of patients with Lyme disease. The most frequently reported clinical manifestation of Lyme carditis is cardiac conduction system disease. The goal of this study was to identify the incidence and predictors of permanent pacemaker implantation in patients hospitalized with Lyme disease.

**Methods:**

A retrospective cohort analysis of the Nationwide Inpatient sample was performed to identify patients hospitalized with Lyme disease in the US between 2003 and 2014. Patients with Lyme carditis were defined as those hospitalized with Lyme disease who also had cardiac conduction disease, acute myocarditis, or acute pericarditis. Patients who already had pacemaker implants at the time of hospitalization (N = 310) were excluded from the Lyme carditis subgroup. The primary study outcome was permanent pacemaker implantation. Secondary outcomes included temporary cardiac pacing, permanent pacemaker implant, and in-hospital mortality.

**Results:**

Of the 96,140 patients hospitalized with Lyme disease during the study period, 10,465 (11%) presented with Lyme carditis. Cardiac conduction system disease was present in 9,729 (93%) of patients with Lyme carditis. Permanent pacemaker implantation was performed in 1,033 patients (1% of all Lyme hospitalizations and 11% of patients with Lyme carditis-associated conduction system disease). Predictors of permanent pacemaker implantation included older age (OR: 1.06 per 1 year; 95% CI:1.05–1.07; P<0.001), complete heart block (OR: 21.5; 95% CI: 12.9–35.7; P<0.001), and sinoatrial node dysfunction (OR: 16.8; 95% CI: 8.7–32.6; P<0.001). In-hospital mortality rate was higher in patients with Lyme carditis (1.5%) than in patients without Lyme carditis (0.5%).

**Conclusions:**

Approximately 11% of patients hospitalized with Lyme disease present with carditis, primarily in the form of cardiac conduction system disease. In this 12-year study, 1% of all hospitalized patients and 11% of those with Lyme-associated cardiac conduction system disease underwent permanent pacemaker implantation.

## Introduction

Lyme disease is the most common tick-borne disease in the United States, Canada, and Europe [1,2]. Previously published studies report that up to 10% of patients hospitalized with Lyme disease exhibit clinical manifestations of the infection of cardiac tissue by Borrelia bacteria [3–9]. These clinical manifestations are commonly referred to in aggregate as Lyme carditis and are identified during the acute disseminated phase of the infection in most patients. The most frequently reported manifestation of Lyme carditis is cardiac conduction system disease (CCD). Less frequently reported cardiac manifestations of Lyme disease include acute myopericarditis and dilated cardiomyopathy [1,2,5,6,10,11].

Atrioventricular (AV) block in the context of Lyme carditis can progress rapidly to complete heart block (CHB). Therefore, patients with Lyme disease who present with evidence of CCD are typically hospitalized and monitored continuously with cardiac telemetry. Although Lyme carditis-related CCD usually resolves with antibiotic therapy, there can be a lag between the initiation of antibiotics and recovery of cardiac conduction. Consequently, some patients require temporary cardiac pacing (TCP) support during the recovery of the cardiac conduction system [3,12,13]. Permanent pacemaker (PPM) implantation is reserved for symptomatic, advanced CCD that persists despite antibiotic treatment [3,10].

The objective of this study was to investigate the impact of Lyme carditis on the outcomes of patients hospitalized with Lyme disease in the US. Multiple outcomes were studied including mortality, heart failure, and PPM implantation.

## Materials and methods

### Query of the National Inpatient Sample database

The data utilized in this study were obtained from the National Inpatient Sample (NIS), the largest collection of all-payer data on inpatient hospitalizations in the United States. The NIS database includes patient- and hospital-level data, including: patient demographics, primary and secondary diagnoses, interventions and procedures, and medical comorbidities (consistent with definitions set forth by the Agency for Healthcare Research and Quality (AHRQ)), and length of stay [14]. For this study, data were obtained from the NIS for the years 2003–2014. Because all NIS datasets are de-identified, this study was exempt from institutional review by the Human Research Committee.

Each unique data entry in the NIS database represents a single hospitalization with a principal discharge diagnosis, secondary diagnosis entries (maximum 29), and procedure entries (maximum 15) [14]. Patients diagnosed with Lyme disease were identified using the International Classification of Diseases, 9th Revision, Clinical Modification (ICD-9-CM) code 088.81.

### Study design

In this study, a patient was defined as having Lyme carditis if they were admitted to an inpatient unit with a diagnosis of Lyme disease during the study period and were also diagnosed with CCD, acute myocarditis, or acute pericarditis. Demographic data were collected for all patients included in the study. Comorbid conditions were identified using measures provided by the AHRQ. Demographic and comorbidity data were used to calculate the Elixhauser comorbidity index for each patient included in the study [15,16]. Patients who underwent TCP or PPM implantation were identified using the appropriate procedure codes. Among patients with CCD, those who had undergone pacemaker implantation prior to the index hospitalization (N = 310) were excluded from this analysis.

### Statistical analysis

Trend weight files were applied in all analyses to account for sampling methods used in the construction of the NIS dataset [17]. All analyses of trends were conducted with the non-parametric test for trends by Cuzick [18].

Baseline characteristics of patients hospitalized with Lyme disease during the study period are displayed in Table 1. Multivariable logistic regression analysis was utilized to identify predictors of patient outcomes, including Lyme carditis, TCP, PPM implantation, and in-hospital mortality. For all analyses, survey estimation was utilized to account for the complex survey design of the NIS database. In all comparisons, a P value of <0.05 was considered significant.

**Table 1:** Trends in baseline characteristics of patients hospitalized with Lyme disease in the U.S. between 2003 and 2014.

	Total	2003	2004	2005	2006	2007	2008	2009	2010	2011	2012	2013	2014	P (Trend)
**Unweighted (N)**	19902	1039	1156	1372	1429	1630	1719	2166	1736	2027	1755	1998	1875	0.005
**Weighted (N)**	96140	4801	5325	6497	7118	7945	8172	10701	8192	9249	8775	9990	9375	0.003
**Median Age in Years (IQR)**	51 (34–66)	49 (33–62)	49 (35–65)	48 (30–63)	49 (32–63)	50 (34–65)	51 (32–65)	51 (35–66)	52 (35–67)	54 (37–67)	52 (34–67)	53 (34–67)	54 (35–68)	0.002
**Gender (%)**														
** Male**	49	48	51	50	50	49	50	48	48	50	50	50	49	0.935
** Female**	51	52	49	50	50	51	50	52	52	50	50	50	51	
**Race (%)**														
** White**	88	88	91	90	89	88	88	89	88	89	86	88	89	0.312
** Black**	4.0	6.0	4.3	3.3	2.4	4.1	3.9	3.1	4.0	4.2	5.1	4.0	4.0	0.870
** Hispanic**	3.9	3.4	3.7	2.8	3.7	4.7	4.6	4.2	5.0	3.4	3.4	3.8	3.4	0.831
** Asian/PI**	1.0	1.8	0.2	0.8	0.4	1.0	1.2	1.0	0.9	0.7	1.3	1.0	1.1	0.441
** Native American**	0.2	0.4	0.2	0.1	0.7	0.5	0.2	0.1	0.3	0.2	0.1	0.1	0.2	0.178
** Others**	2.6	0.9	1.0	3.0	3.5	1.6	2.6	2.2	1.9	2.7	3.9	3.6	2.5	0.086
**Comorbidities (%)**														
** Hypertension**	32	21	27	26	27	30	30	32	35	37	35	35	36	0.003
** CHF**	2.8	1.8	2.7	1.9	1.7	2.5	2.8	2.5	3.7	3.3	3.3	3.1	3.4	0.009
** PVD**	2.2	1.0	1.8	1.4	1.7	2.0	1.6	2.5	2.4	2.6	2.6	2.6	2.9	0.002
** DM**	11	5.4	7.7	8.2	8.2	11	10	11	11	12	12	12	13	0.001
** CKD**	3.1	0.7	0.9	1.0	1.9	2.2	2.7	3.5	3.9	4.4	3.9	4.4	4.1	0.002
** CPD**	12	8.0	11	11	11	12	12	11	11	13	13	13	15	0.003
**Elixhauser Score (%)**		** **												
** <3**	65	78	69	70	70	69	67	62	64	63	62	60	59	0.002
** ≥3**	35	22	32	30	30	31	33	38	36	37	38	40	41	
**Lyme Carditis (%)**	11	9.5	8.7	10	10	10	8.8	11	11	12	12	12	12	0.004
** Acute Myocarditis**	2.1	1.6	1.3	2.3	1.7	1.9	2.1	2.5	2.5	2.0	2.1	2.6	1.8	0.092
** Acute Pericarditis**	0.3	0.5	0.2	0.1	0.1	0.5	0.2	0.3	0.2	0.3	0.2	0.2	0.4	0.744
** Conduction Disease**	10	8.7	8.1	9.5	9.1	9.7	8.0	11	9.8	11	12	11	11	0.007
** TCP**	1.1	0.8	1.1	0.7	1.2	1.2	1.0	0.9	1.3	1.2	1.0	1.1	1.1	0.322
** PPM**	1.1	1.2	1.3	1.0	1.1	1.5	0.8	1.1	0.7	1.2	1.1	0.8	1.1	0.209
**Mortality (%)**	0.6	0.3	1.2	0.5	0.3	0.6	0.4	0.5	0.6	0.9	1.0	0.7	0.7	0.115
** Mean LOS (Days)**	4.7	4.4	4.8	4.5	4.4	4.8	4.6	4.6	4.8	4.7	4.7	4.7	4.9	0.089

CHF: Congestive heart failure; PVD: Peripheral vascular disease; VD: Valvular disease; DM: Diabetes mellitus; CKD: Chronic Kidney disease; CPD: Chronic pulmonary disease; LOS: Length of stay.

All analyses were completed using Stata/SE software version 14.1 (StataCorp LP, College Station, Texas).

## Results

### Baseline characteristics

**Fig 1:**
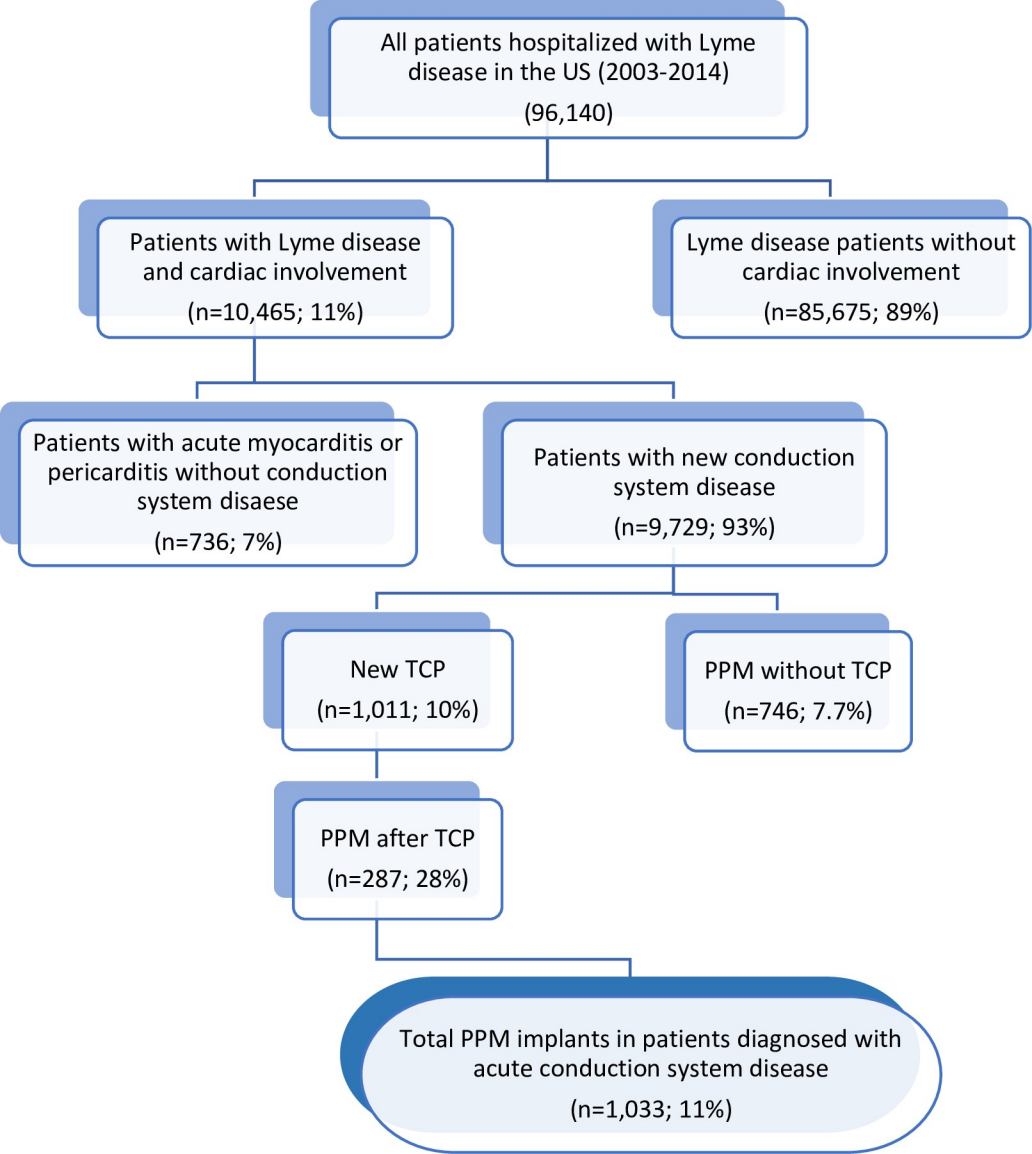
Proportion of patients hospitalized with Lyme disease who underwent implant of permanent pacemakers. Flow chart showing the total number of patients hospitalized with Lyme disease in the US during the study period. Also displayed is the number of patients with cardiac issues related to Lyme disease, including: myocarditis, pericarditis, and conduction system disease (CCD). Some patients with Lyme-related CCD underwent implant of a permanent pacemaker (PPM) after implant of a temporary cardiac pacemaker (TCP). Other patients with Lyme-related CCD underwent PPM implant without first undergoing TCP implant.

A total of 96,140 patients were hospitalized with Lyme disease during the study period (Fig 1). The baseline characteristics of patients hospitalized with Lyme disease in the United States between 2003 and 2014 are summarized in Table 1. There was a near-equal distribution of females and males (51% and 49%, respectively) in the study population. The median (IQR) age was 51 (34–66) years. Most patients in this study were white (88%).

The most prevalent comorbidity among patients hospitalized with Lyme disease was hypertension (32%). Other commonly reported comorbidities included chronic lung disease (12%) and diabetes mellitus (11%). A significant increase in the prevalence of all comorbidities except valvular disease was observed between 2003 and 2014 (Table 1). This increase was also evident in the Elixhauser score, as the number of patients with an Elixhauser score greater than 2 increased from 22% in 2003 to 41% in 2014 (P trend = 0.002).

### Temporal and regional trends in Lyme disease-related hospitalizations

There was a significant increase in the number of patients hospitalized with Lyme disease during the study period, with a near-doubling from 4,801 in 2003 to 9,375 patients in 2014 (P trend = 0.003). A comparable difference was observed among patients with Lyme carditis (Table 2). A majority of the hospitalizations for Lyme disease occurred in the Northeast region of the United States (61%). The South, Midwest, and West accounted for 22%, 13%, and 5% of hospitalizations, respectively.

**Table 2:** Trends in baseline characteristics of patients hospitalized with Lyme carditis in the U.S. between 2003 and 2014.

	Total	2003	2004	2005	2006	2007	2008	2009	2010	2011	2012	2013	2014	P (Trend)
**Unweighted (N)**	2156	98	102	142	142	167	150	246	188	242	218	242	227	0.005
**Weighted (N)**	10465	458	464	666	709	825	717	1203	880	1108	1090	1210	1135	0.003
**Median Age in Years (IQR)**	52 (34–68)	43 (31–65)	49 (37–63)	48 (31–65)	52 (35–65)	50 (36–66)	50.5 (32–68)	48 (32–66)	52.5 (35.5–72)	56 (38–72)	55 (37–73)	56 (31–69)	54 (34–70)	0.007
**Gender (%)**														
** Male**	62	65	66	65	67	55	62	61	59	63	63	58	65	0.228
** Female**	38	35	34	36	33	45	38	39	42	37	37	42	35	
**Race (%)**														
** White**	87	84	91	90	86	84	82	88	85	92	86	88	88	0.609
** Black**	4.5	7.5	4.5	3.1	4.4	7.9	3.8	4.0	4.9	3.4	6.3	2.7	4.1	0.256
** Hispanic**	3.8	2.4	4.5	2.9	1.8	5.6	7.1	4.6	3.7	3.8	2.9	3.1	3.2	0.736
** Asian/PI**	1.0	1.1	0.0	0.0	1.0	0.7	3.0	0.9	1.8	0.0	1.9	0.4	1.4	0.375
** Native American**	0.5	0.0	0.0	0.0	3.1	1.3	0.0	0.0	1.0	0.0	0.0	0.0	0.9	0.901
** Others**	3.1	4.7	0.0	4.0	3.3	1.0	4.5	2.8	3.3	1.3	3.4	5.8	2.3	0.944
**Comorbidities (%)**														
** Hypertension**	35	21	28	26	35	29	35	31	37	41	38	40	44	0.002
** CHF**	4.4	4.5	3.2	3.8	2.1	5.9	6.1	3.1	6.5	6.0	2.3	4.1	4.8	0.531
** PVD**	3.3	0.0	3.9	1.3	2.0	2.3	3.5	2.9	3.3	4.4	3.2	4.5	5.3	0.016
** DM**	11	0.9	4.0	8.7	7.4	8.2	15	8.7	10	12	13	15	15	0.006
** CKD**	4.3	0.9	0.0	0.0	4.1	1.7	3.3	2.5	5.4	7.3	8.3	4.5	6.6	0.005
** CPD**	11	4.4	9.0	7.8	9.7	7.8	10	14	9.3	11	12	12	15	0.005
**Elixhauser Score (%)**														
** <3**	66	80	75	74	69	69	72	69	65	62	60	65	53	0.003
** ≥3**	34	20	25	26	32	31	28	31	36	38	40	35	47	
**Acute Myocarditis**	19	17	15	23	17	18	24	22	23	17	17	21	15	0.835
**Acute Pericarditis**	2.5	5.2	2.6	0.7	1.3	4.8	2.5	2.6	2.1	2.5	1.8	1.7	3.5	0.569
**Conduction Disease**	93	91	93	93	92	93	92	95	91	95	93	93	93	0.378
**Mortality (%)**	1.5	0.9	1.0	0.8	0.7	1.8	0.6	2.1	0.5	1.6	3.7	1.2	1.8	0.153
**Mean LOS (Days)**	5.3	4.7	4.4	5.2	5.1	5.8	4.6	5.4	5.6	5.4	5.1	5.3	5.6	0.084

CHF: Congestive heart failure; PVD: Peripheral vascular disease; VD: Valvular disease; DM: Diabetes mellitus; CKD: Chronic Kidney disease; CPD: Chronic pulmonary disease; LOS: Length of stay.

### Lyme carditis in patients hospitalized with Lyme disease

Patients with Lyme carditis constituted 11% (N = 10,465) of all patients hospitalized with Lyme disease during the study period (Fig 1). New CCD was the most common manifestation of Lyme carditis and was observed in 10% of patients hospitalized with Lyme disease during the study period (93% of patients with Lyme carditis). Acute myocarditis and acute pericarditis were rare and comprised 2.1% and 0.3% of all patients hospitalized Lyme disease, respectively.

A majority of patients with Lyme carditis were male (62%, Table 2). The median (IQR) age was 52 (34–68) years. A majority of patients with Lyme carditis were hospitalized in urban teaching hospitals (52%), while hospitalizations with Lyme carditis in urban non-teaching and rural hospitals constituted 37% and 11%, respectively. Most patients with Lyme carditis were white (87%) and most Lyme carditis-related hospitalizations were in the Northeast region (65%), while the South, Midwest, and West accounted for 20%, 11% and 4% of hospitalizations, respectively.

Several medical comorbidities were observed more frequently in patients with Lyme carditis than in the general population of patients with Lyme disease. These include hypertension (35% vs 32%; P<0.001), congestive heart failure (4.4% vs 2.8%; P<0.001), peripheral vascular disease (3.3% vs 2.2%; P<0.001), and chronic kidney disease (4.3% vs 3.1%; P<0.001).

### Clinical manifestations of Lyme carditis in hospitalized patients

Among the 10,465 patients hospitalized with Lyme disease who also had evidence of Lyme carditis, 9,729 (93%) were diagnosed with CCD. Acute myocarditis was also reported in 2,002 (19%) patients and acute pericarditis in 264 (2.5%) patients diagnosed with Lyme carditis. Among patients diagnosed with CCD, first-degree AV block was present in 1,068 (11%), second degree AV block in 1,334 (14%), complete AV block in 2,282 (23%), and unspecified AV block in 59 (0.6%) cases (Table 3). Right bundle branch block (RBBB) and left bundle branch block (LBBB) were present in 755 (7.8%) and 596 (6.1%) patients with conduction system disease, respectively. Other bundle branch blocks (including bifascicular and trifascicular block) were present in 236 (2.4%) patients, and the type of bundle branch block was unspecified in 22 (0.2%) cases. Sino-atrial (SA) node dysfunction was diagnosed in 569 (5.8%) patients, and cardiac arrest occurred in 301 (3.1%) patients. Unspecified CCD was reported in 3,654 (38%) patients.

**Table 3:** Conduction system disease and pacemaker implantation in patients hospitalized with Lyme carditis in the U.S. between 2003 and 2014.

Conduction System Disease	Total	TCP	PPM after TCP	PPM without TCP	Total PPM	Mortality
**1° AV Block, N (%)**	**1068 (11)**	83 (7.8)	5 (6)	53 (5.4)	58 (5.4)	0
**2° AV block, N (%)**	**1334 (14)**	148 (11)	24 (16)	91 (7.7)	115 (8.6)	19 (1.4)
**Complete AV Block, N (%)**	**2282 (24)**	833 (37)	221 (27)	383 (26)	604 (27)	14 (0.6)
**Unspecified AV Block, N (%)**	**59 (0.6)**	9 (15)	4 (48)	5 (10)	9 (16)	0
**Right BBB, N (%)**	**755 (7.8)**	50 (6.6)	15 (31)	34 (4.8)	49 (6.5)	5 (0.7)
**Left BBB, N (%)**	**596 (6.1)**	39 (6.6)	19 (50)	42 (7.5)	61 (10)	4 (0.7)
**Other BBB, N (%)**	**236 (2.4)**	38 (16)	23 (60)	20 (10)	44 (19)	5 (2.1)
**Unspecified BBB, N (%)**	**22 (0.2)**	4 (20)	4 (100)	0 (0)	4 (20)	0
**SA node dysfunction, N (%)**	**569 (5.8)**	89 (15.7)	56 (62)	241 (50)	297 (52)	10 (1.8)
**Unspecified CD, N (%)**	**3654 (38)**	14 (0.4)	10 (68)	51 (1.4)	60 (1.7)	45 (1.2)
**Cardiac arrest, N (%)**	**301 (3.1)**	101 (34)	53 (53)	14 (7.2)	68 (23)	67 (22)
**Total, N (%)**	**9729 (100)**	**1011 (10)**	**287 (28)**	**746 (7.7)**	**1033 (11)**	**155 (1.6)**

AVB: Atrioventricular block; BBB: Bundle branch block; SA: Sino-atrial; CD: Conduction disease.

### Predictors of Lyme carditis

Predictors of Lyme carditis in patients hospitalized with Lyme disease include older age (OR: 1.004 per 1 year; 95% CI: 1.002–1.007; P = 0.001), male sex (OR: 1.7; 95% CI: 1.6–1.9; P<0.001), peripheral vascular disease (OR: 1.3; 95% CI: 1.0–1.7; P = 0.033), valvular disease (OR: 1.7; 95% CI: 1.4–2.2; P<0.001), chronic pulmonary disease (OR: 1.7; 95% CI: 1.2–2.5; P = 0.006), Black race (OR: 1.3; 95% CI: 1.01–1.6; P = 0.041), and Native American race (OR: 2.6; 95% CI: 1.3–5.3; P = 0.007) compared to whites (Table 4).

**Table 4:** Multivariable logistic regression: Predictors of Lyme carditis in patients hospitalized with Lyme disease in the U.S. between 2003 and 2014.

Variables	OR*	95% CI	P value
**Age**	1.004	1.002–1.007	0.001
**Male Sex**	1.7	1.6–1.9	0.000
**PVD**	1.3	1.0–1.7	0.033
**VD**	1.7	1.4–2.2	0.000
**CPD**	1.7	1.2–2.5	0.006
**Race**	Ref: White		
** Black**	1.3	1.0–1.6	0.041
** Hispanic**	1.1	0.9–1.4	0.457
** Asian/PI**	1.1	0.7–1.8	0.645
** Native American**	2.6	1.3–5.3	0.007
** Others**	1.2	0.9–1.6	0.164

CI: Confidence interval; PVD: Peripheral vascular disease; VD: Valvular disease; CPD: Chronic pulmonary disease; PI: Pacific Islander; OR*: Odds ratios adjusted for sex, income status, hospital region, hospital teaching status, hospital bed size, and calendar year.

### Pacemaker implantation in patients with Lyme carditis

**Fig 2:**
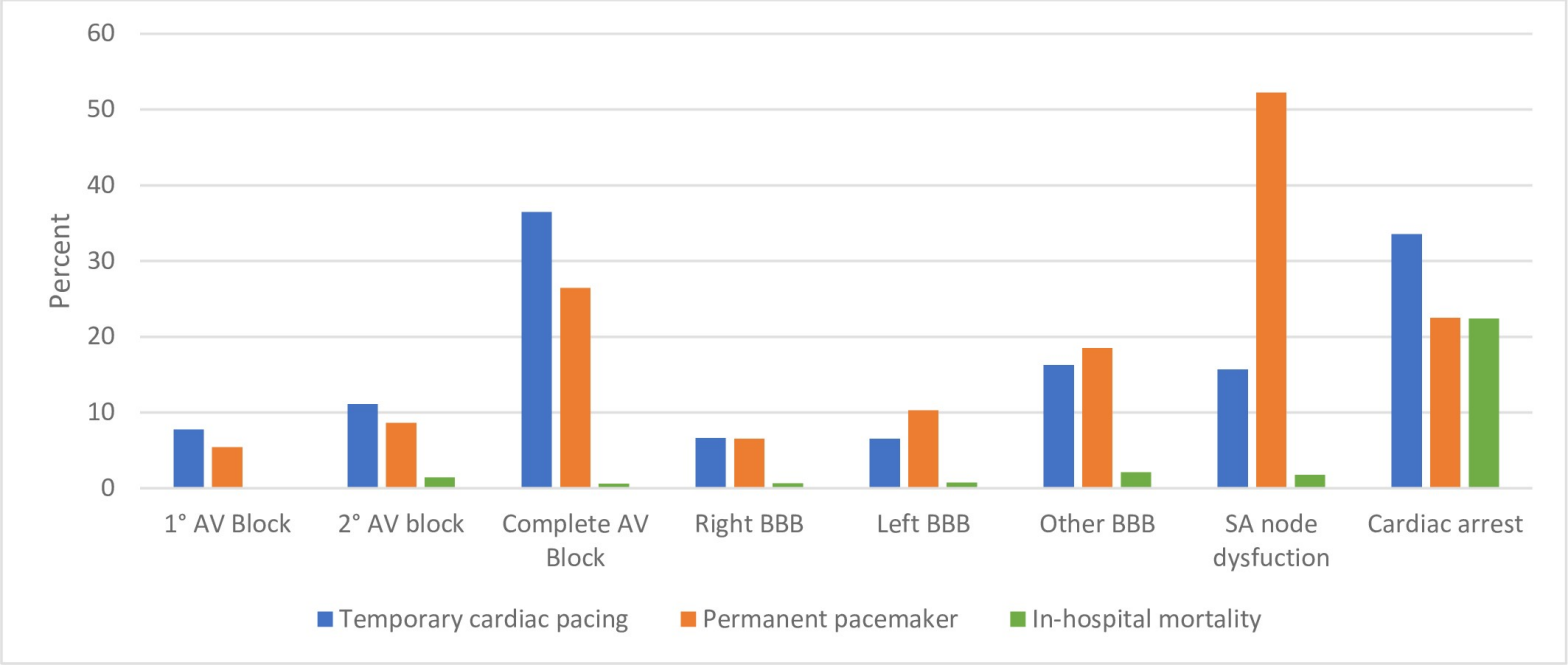
Pacemaker implant and mortality rates stratified by conduction disease. Bar graph displaying the rates of temporary/permanent pacemaker implant and in-hospital mortality for each type of heart rhythm disorder described in this study.

Outcomes of patients hospitalized with Lyme disease who also had evidence of Lyme carditis are summarized in Table 5. Of the 9,729 patients diagnosed with CCD, 1,011 (10%) underwent TCP, of whom 287 (28%) ultimately underwent PPM implantation (Fig 1). Among patients with evidence of CCD, 746 (8%) underwent PPM implantation without first undergoing TCP. Overall, 11% of patients with Lyme carditis-associated CCD (N = 1,033) underwent PPM implantation. A majority of the patients (N = 833, 82%) who underwent TCP had a diagnosis of complete AV block (Fig 2). SA node dysfunction, first-degree AV block and second-degree AV block were present in 9%, 8%, and 15% of patients who underwent TCP implantation, respectively. Among patients who underwent PPM implantation, 604 (58%) were diagnosed with complete AV block. SA node dysfunction, first-degree AV block, and second-degree AV block were present in 29%, 6%, and 11% of patients, respectively.

**Table 5:** Outcomes in patients hospitalized with Lyme carditis.

Outcome	N (%)	P value
**Lyme Carditis**	**10465 (100)**	
Acute Myocarditis/Pericarditis*	736 (7)	
Conduction System Disease	9729 (93)	
**Conduction System Subgroup**		
** **Temporary Cardiac Pacing	1011 (10.4)	
** **PPM after TCP	287 (28.4)	
** **PPM without TCP	746 (7.7)	
** **All Permanent Pacemaker	1033 (10.6)	
**Acute Heart Failure**		
** **Total Population	825 (0.86)	
** **Lyme Carditis	248 (2.37)	
** **Lyme Disease without Carditis	577 (0.67)	**<0.001**
**Cardiogenic Shock**		
** **Total Population	125 (0.13)	
** **Lyme Carditis	48 (0.46)	
** **Lyme Disease without Carditis	76 (0.09)	**<0.001**
**In-Hospital Mortality**		
** **Total Population	613 (0.64)	
** **Lyme Carditis	161 (1.54)	
** **Lyme Disease without Carditis	453 (0.53)	**<0.001**
**Mean Length of Stay (Days)**		
** **Total Population	4.7	
** **Lyme Carditis	5.3	
** **Lyme Disease without Carditis	4.7	**<0.001**

*Acute myocarditis or pericarditis in the absence of cardiac conduction system disease.

### Impact of Lyme carditis on cardiovascular adverse events and in-hospital mortality

The incidence of acute heart failure, cardiogenic shock, and death among patients hospitalized with Lyme disease was 0.9%, 0.1%, and 0.6%, respectively. Acute heart failure occurred in 2.4% of patients with Lyme carditis. This was approximately four-fold higher than the incidence of acute heart failure in patients without Lyme carditis (0.7%; P<0.001). In addition, the incidence of cardiogenic shock in patients with Lyme carditis was five-fold higher than that in patients without Lyme carditis (0.5% vs 0.1; P<0.001). The mean length of stay for patients with Lyme carditis was 5.2 days compared to 4.6 days (P<0.001) among those without cardiac involvement. The in-hospital mortality rate among patients with Lyme carditis was 1.5% compared with 0.5% among patients without Lyme carditis (P<0.001). Independent predictors of in-hospital mortality among patients with Lyme carditis (Table 6) include older age (OR: 1.03 per 1 year; 95% CI: 1.004–1.06; P = 0.025), Elixhauser score >2 (OR: 5; 95% CI: 1.6–15.1; P = 0.005), and cardiac arrest (OR: 54.2; 95% CI: 15.4–191.3; P<0.001).

**Table 6:** Multivariable logistic regression: Predictors of TCP, PPM implantation, and mortality in the U.S. between 2003 and 2014.

	TCP			PPM			In-Hospital Mortality		
Variables	OR*	95% CI	P value	OR*	95% CI	P value	OR*	95% CI	P value
**Age**	1.0	0.98–1.00	0.081	1.06	1.05–1.07	<0.001	1.0	1.004–1.06	0.025
**Female Sex**	0.7	0.5–1.1	0.123	1.2	0.8–1.7	0.462	1.7	0.6–4.6	0.312
**Elixhauser Score >2**	0.9	0.5–1.4	0.493	0.7	0.4–1.0	0.058	5.0	1.6–15.1	0.005
**Second Degree AVB**	2.0	1.1–3.6	0.020	1.4	0.7–2.6	0.372	2.3	0.6–9.8	0.253
**Complete AVB**	33.0	20.3–53.7	<0.001	21.45	12.9–35.7	<0.001	0.2	0.04–1.3	0.092
**Cardiac Arrest**	8.8	385–20.5	<0.001	2.9	1.3–6.8	0.013	54.2	15.4–191.3	<0.001
**SA Node Dysfunction**	5.2	2.1–13.0	<0.001	16.8	8.7–32.6	<0.001	1.3	0.3–5.6	0.742
**Unspecified AVB**	7.5	1.6–36.8	0.013	2.7	0.4–20.0	0.342	1.0	-	-
**Unspecified BBB**	8.1	1.6–41.4	0.012	2.7	0.6–12.7	0.22	1.0	-	-

CI: Confidence interval; AVB: Atrioventricular block; SA: Sino-atrial; CCD: Cardiac conduction system disease; OR*: Odds ratios adjusted for sex, race, income status, hospital region, hospital teaching status, hospital bed size, and calendar year.

### Predictors of pacemaker implantation in patients with Lyme carditis

The results of the multivariable logistic regression analysis to determine the predictors of TCP, PPM implantation, and in-hospital mortality in patients hospitalized with Lyme carditis are shown in Table 6. The odds ratios (OR) were adjusted for gender, race, calendar year, median household income, hospital size/type, and region. The strongest predictors of TCP in patients with Lyme carditis include complete AV block (OR: 33; 95% CI: 20.3–53.7; P<0.001) and cardiac arrest (OR: 8.8; 95% CI: 3.8–20.5; P<0.001). Other predictors of TCP include second degree AV block (OR: 2.0; 95% CI: 1.1–3.6; P = 0.02), SA node dysfunction (OR: 5.2; 95% CI: 2.1–13; P<0.001), unspecified AV block (OR: 7.5; 95% CI: 1.6–36.8; P = 0.013), and unspecified BBB (OR: 8.1; 95% CI: 1.6–41.4; P = 0.012).

Among patients with CCD, older patients were more likely to undergo PPM implantation (OR: 1.06 per 1 year; 95% CI:1.05–1.07; P<0.001). Other predictors of PPM implantation include complete heart block (OR: 21.5; 95% CI: 12.9–35.7; P<0.001), cardiac arrest (OR: 2.9; 95% CI: 1.3–6.8; P = 0.013) and SA node dysfunction (OR: 16.8; 95% CI: 8.7–32.6; P<0.001).

## Discussion

This study utilized the largest all-payer inpatient database in the United States to assess cardiovascular outcomes in patients hospitalized with Lyme disease over the 12-year period between 2003–2014. During the study period, the number of patients hospitalized annually with Lyme disease more than doubled. Our data revealed that among hospitalized patients 11% exhibited evidence of Lyme carditis, a large majority of whom were diagnosed with CCD (Fig 1). Among patients with Lyme carditis-associated CCD, 11% underwent PPM implant during the index hospitalization. This represents 1% of all patients hospitalized with Lyme disease. The independent predictors of PPM implantation include increased age, presence of CHB, SA node dysfunction, and cardiac arrest.

Estimates based on insurance claims data suggest that approximately 329,000 individuals in the U.S. were diagnosed with and treated for Lyme disease annually between 2005 and 2010. This estimate increased to 476,000 annually between 2010 and 2018 [19–21]. A similar increase in Lyme disease cases was reported in Canada [22]. This trend has been attributed to increased prevalence of Lyme disease. These findings likely explain the increasing numbers of hospitalizations for Lyme disease and Lyme carditis between 2003–2014 observed in this study. The increase in the prevalence of some comorbidities during the study period may be attributable a least in part to aging of the population.

Our study found that older age and male sex were associated with higher odds of developing Lyme carditis in patients with Lyme disease (Table 4) [19]. The association between age and Lyme carditis is likely driven by multiple factors. Although our study revealed that several age-related comorbidities (e.g., vascular disease and chronic pulmonary disease) are independent predictors of Lyme carditis, it is possible that other factors that we could not measure (e.g., pre-existing, subclinical cardiac conduction system disease) contributed as well. Several explanations for sex-related differences have been proposed, including differences in health-seeking behavior and differential susceptibility to non-cutaneous forms of Lyme disease [10,23].

Lyme carditis is the result of an inflammatory response to the invasion of cardiac tissue by Borrelia burgdorferi. The signs and symptoms of Lyme carditis typically resolve with antibiotic treatment [24–28]. Even so, Lyme carditis is associated with rare but serious adverse cardiovascular outcomes, including congestive heart failure, cardiogenic shock, and sudden cardiac death [8,10,26,29–36]. This study demonstrated that for patients hospitalized with Lyme disease, the presence of Lyme carditis was associated with a four-fold increase in the incidence of acute heart failure (2.4% vs 0.7%), a five-fold increase in the incidence of cardiogenic shock (0.5% vs 0.1%) and a three-fold increase in hospital mortality rate (1.5% vs 0.5%).

In this study, approximately 10% of patients hospitalized with Lyme disease and CCD underwent TCP. The rate of TCP implant was highest in patients with CHB (37%). More than 1 in 4 patients (28%) in our study who underwent TCP subsequently received PPM implants. Of the patients in this study with Lyme carditis who were diagnosed with CCD, 7.7% received PPM implant without first undergoing TCP. A majority of these patients were diagnosed with advanced CCD.

Guidelines for antibiotic treatment of Lyme disease are well-validated, however, data relevant to the management of conduction system disease in Lyme carditis are limited to case reports and small single-center studies [6,8,10]. Consequently, there are no formal, evidence-based guidelines for pacemaker therapy in patients with Lyme disease who present with CCD. Recent guidelines for pacemaker therapy in patients with CCD state that PPM implant is contraindicated in the context of acute Lyme carditis (Class III indication) as the AV block is expected to resolve without recurrence [37]. Although a large majority of cases of Lyme carditis resolve within two weeks of antibiotic treatment, CCD may persist for up to 6 weeks and beyond. Some reports have shown that Lyme carditis can cause persistent AV block even with adequate antimicrobial therapy [2,4,30,38–41]. This is one possible explanation for the range of rates reported for PPM implantation in patients with Lyme carditis: 10% in our study and 4–13% in other published studies [10,22,41].

The use of temporary transvenous pacemakers is usually limited to few days, and requires immobilization and continuous telemetry monitoring, with risk for dislodgement, infection or cardiac perforation [42]. The use of temporary-permanent pacemakers (TPP), has been proposed as a strategy to reduce the rate of PPM implant rate in cases of Lyme carditis where cardiac conduction system recovery is prolonged [22,43–45]. However, there are only a few published reports of TPP use in patients with Lyme carditis, and further investigation is required [42,43].

It is possible that longitudinal monitoring of patients with Lyme disease and persistent conduction system abnormalities may reduce the rate of PPM implantation. The “Suspicious index in Lyme carditis” (SILC) score, which was described after the start of the time period included in our study, uses key risk factors for Lyme carditis to establish Lyme disease as the etiology of CCD [41]. Application of this score in patients with CCD may help prompt the initiation of antibiotic therapy and reduce the number of patients who undergo PPM implantation. Retrospective application of the SILC score showed that 55% of patients who underwent PPM implantation due to Lyme carditis-related AV block eventually exhibited resolution of the underlying conduction disease [41]. However, the SILC score does not provide guidelines for the use of PPM implant in patients with CCD confirmed to be due to Lyme disease. Our study was not designed to assess the impact of a systematic approach such as the SILC score to the diagnosis and treatment of Lyme carditis on PPM utilization in this patient population [22].

Limitations of the NIS dataset prevented us from performing a comprehensive analysis of the appropriateness of PPM implant in patients with Lyme carditis. The NIS dataset does not include data regarding the number of patients who exhibited resolution of CCD during hospital admission. In addition, the NIS does not provide outcomes data after the index hospitalization. For those patients who underwent PPM implant, the NIS dataset does not provide information on the percentage of time that pacing was utilized after device implantation. Thus, the percentage of patients with Lyme carditis in whom pacemaker implantation might have been safely avoided cannot be assessed with this database.

### Limitations

Recognized limitations of the NIS database include potential errors in coding of diagnoses. Not all listed diagnoses are associated with a time of onset and resolution, limiting the ability to determine the sequence of events in some cases. Each NIS entry corresponds to a single hospitalization and transfer between hospitals can produce more than one entry for an individual patient. This study included only hospitalized patients and the results cannot be generalized to the outpatient population. In addition, the NIS does not contain post-hospitalization data. Therefore, it was not possible to analyze the number of patients in whom Lyme carditis-related CCD resolved with treatment. The NIS database also does not include data on the utilization of cardiac pacing in those patients who underwent PPM implantation. Furthermore, post-hospitalization outcomes of patients with and without Lyme carditis could not be assessed with this database.

## Conclusions

Lyme carditis is present in 11% of patients hospitalized with Lyme disease in the U.S. In this study, CCD was present in 93% of patients with Lyme carditis. Patients hospitalized with Lyme carditis have a four-fold higher incidence of acute heart failure, a five-fold higher incidence of cardiogenic shock, and a three-fold higher mortality rate compared to patients without cardiac involvement. Among patients with Lyme carditis and associated CCD, 10% underwent TCP and 11% underwent PPM implantation. In addition, 7.7% of patients with CCD underwent PPM implantation without first undergoing TCP.
